# Interfacial Molecular Behavior and Organization of Low-Fluorine Surfactants at the sc-CO_2_/Water Interface

**DOI:** 10.3390/molecules31193576

**Published:** 2026-10-08

**Authors:** Chunlong Xue, Sihan Feng, Shan Gao, Chenkang Zhao, Hongyu Guo, Xiaorong Li, Guanghui Zhao, Dongjun Wang, Yanfeng Liu, Ying Li

**Affiliations:** 1 Daqing Petrochemical Research Center, Petrochemical Research Institute of PetroChina, Daqing 163714, China; 2 Key Lab. of Colloid and Interface Chemistry of State Education Ministry, School of Chemistry and Chemical Engineering, Shandong University, Jinan 250100, China

**Keywords:** low-fluorinated surfactant, sc-CO_2_/water interface, molecular dynamics simulation, molecular interfacial behavior

## Abstract

Understanding the molecular organization of surfactants at the sc-CO_2_/water interface is essential for the rational design of CO_2_-philic interfacial formulations with reduced fluorine content. In this work, three low-fluorinated anionic surfactants (LF1–LF3) with different hydrophobic-tail architectures were investigated together with a fluorine-free branched co-surfactant (ZBJ) using density functional theory (DFT) calculations and molecular dynamics (MD) simulations. DFT calculations revealed a clear amphiphilic segregation of solvent affinity: the fluorinated tails preferentially interacted with CO_2_, whereas the sulfonate headgroups exhibited stronger affinity for water. MD simulations showed that the surfactants, initially positioned at the sc-CO_2_/water interface, underwent interfacial reorganization and orientational ordering, with their fluorinated tails extending into the sc-CO_2_ phase and their hydrophilic headgroups oriented toward the aqueous phase. Tail branching strongly affected interfacial packing and structural stability; the three-tailed, claw-like LF3 formed the thickest and most thermodynamically stable monolayer, with an interfacial formation energy of −330.30 kJ/mol. In the LF3/ZBJ mixed system, ZBJ occupied packing voids between LF3 molecules, further stabilizing the mixed monolayer and yielding an interfacial formation energy of −342.07 kJ/mol. These results clarify how tail architecture, CO_2_-philic/hydrophilic partitioning, counterion coordination, and steric complementarity govern the interfacial organization and stabilization of low-fluorine surfactants, providing molecular-level guidance for the rational design of low-fluorine surfactant formulations for sc-CO_2_/water systems.

## 1. Introduction

Unlike the marine-dominated tight oil reservoirs in North America, continental tight oil reservoirs, heavily represented in China, pose unparalleled development challenges due to their ultra-low permeability, low rock brittleness, poorly developed natural fractures, low pressure coefficients, and high crude viscosity [1,2]. These unfavorable geological features seriously impede conventional hydraulic fracturing, which furthermore suffers from excessive freshwater consumption and severe environmental footprints [3,4]. Supercritical carbon dioxide (sc-CO_2_) fracturing has emerged as a revolutionary, waterless alternative, offering minimal formation damage, high proppant conductivity, and enhanced oil recovery (EOR) benefits [5]. However, the intrinsic low viscosity and density of sc-CO_2_ lead to severe proppant settling, excessive fluid loss, narrow fracture widths, and detrimental viscous fingering or gravity overriding [6]. Consequently, thickening sc-CO_2_ is a non-negotiable prerequisite for its widespread industrial application.

To mitigate these limitations, incorporating sc-CO_2_-soluble thickeners, including polymers, small molecules, and surfactants, have been extensively explored [7,8,9]. While polymers effectively increase viscosity, their harsh synthesis routes and prohibitive costs restrict commercial viability [10]. Surfactants capable of self-assembling into viscosity-enhancing reversed micelles or rod-like aggregates within CO_2_ offer a promising alternative [11]. In particular, fluorinated surfactants have garnered significant attention, as their highly polarized fluorocarbon tails possess exceptional CO_2_-philicity, enabling the formation of robust associative networks that elevate apparent viscosity [12]. Nevertheless, conventional perfluorinated surfactants are severely constrained by environmental persistence (bioaccumulation) and high cost. Minimizing the fluorine content—by engineering partially fluorinated hydrocarbon surfactants that preserve stable sc-CO_2_ affinity and interfacial activity-represents a critical yet challenging pathway to overcome this eco-economic bottleneck [13]. Reducing the degree of fluorination through partially fluorinated molecular architectures therefore represents a promising strategy for decreasing the overall use of fluorinated moieties while attempting to retain the CO_2_-philicity and interfacial activity required for sc-CO_2_ applications [14,15]. However, partial fluorination does not necessarily eliminate environmental persistence, because C-F bonds remain present in these molecules. Accordingly, the potential environmental benefit of low-fluorine surfactants should be evaluated in terms of the actual reduction in fluorine content, together with their long-term persistence, biodegradability, and ecotoxicity [16].

Elucidating the molecular-level behavior and structure–activity relationships of these surfactants under harsh reservoir conditions is paramount for rational design, yet experimental diagnostics remain exceptionally difficult. Molecular dynamics (MD) simulations have recently provided insights into the interfacial behavior of fluorinated surfactants, revealing that strong electronic dipoles in C-F bonds govern CO_2_-philicity [17,18]. However, a systematic understanding of the subtle interactions between cost-effective, low-fluorine surfactants and sc-CO_2_ remains noticeably absent [19,20]. More importantly, how low-fluorine surfactants reorganize, pack, and orient within the sc-CO_2_/water interfacial layer, as well as how their molecular architecture governs the compactness and stability of the resulting interfacial film, remains insufficiently understood [21].

To address these critical gaps, this work systematically designs a series of low-fluorinated hydrocarbon surfactants featuring tailored hydrophobic tail architectures with minimal fluorine atom substitution. First, density functional theory (DFT) calculations were performed to map the molecular electrostatic potentials (ESP) and quantify the binding energies of these variants with CO_2_ and H_2_O. Subsequently, MD simulations were employed to investigate interfacial reorganization, molecular packing, and orientational ordering at the sc-CO_2_/water interface and to characterize the microstructure of the sc-CO_2_-water-surfactant associative systems. The objective is to identify molecular interactions relevant to thickener design. This comprehensive approach establishes a structural relationship linking tail branching, low-fluorination, and interfacial organization, providing molecular-level guidance for designing the next generation of low-fluorine sc-CO_2_ formulations.

## 2. Results

### 2.1. DFT Analysis of Low-Fluorinated Surfactants

**Figure 1:**
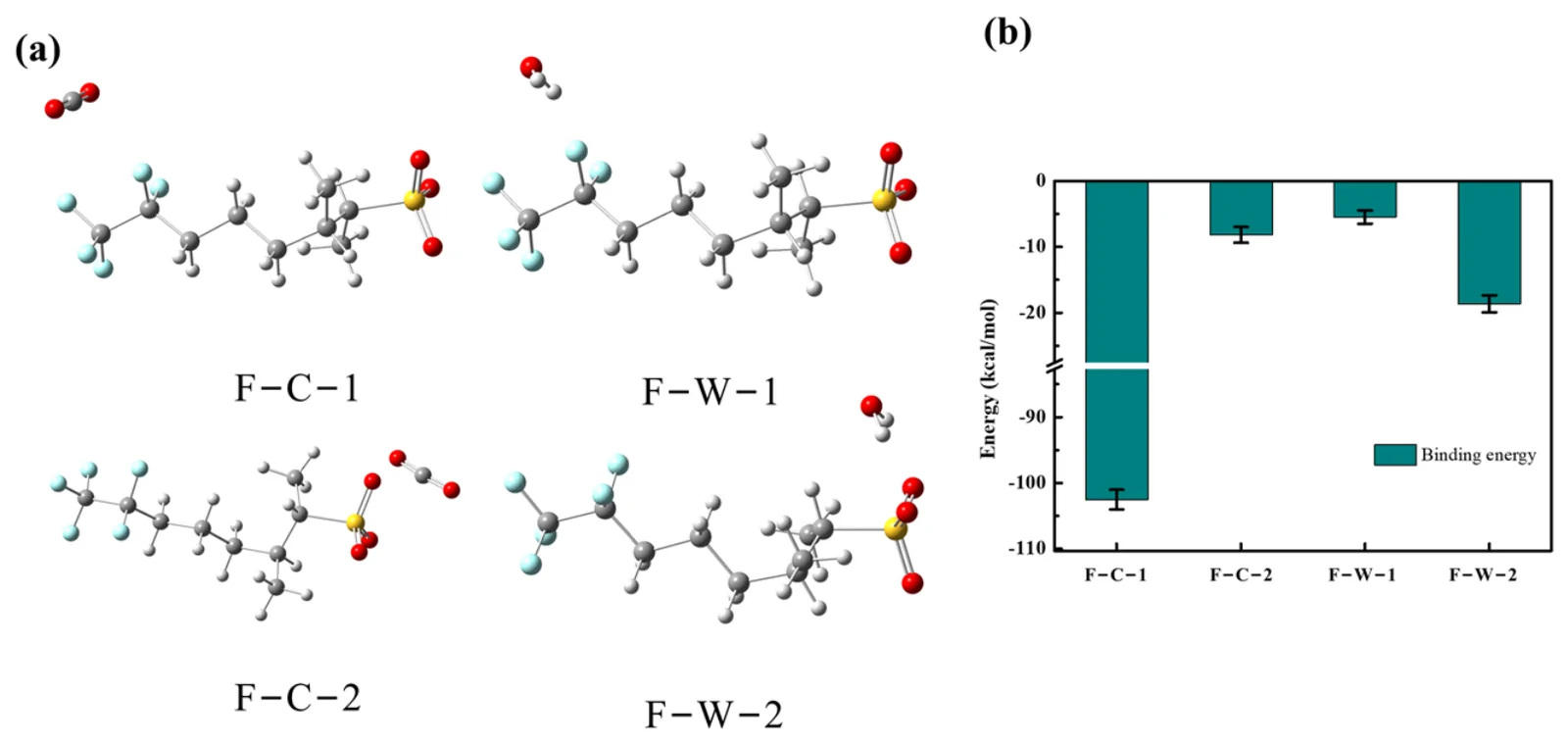
(**a**) Optimized configurations of CO_2_/water adsorbed on LF1. Atom colors in the DFT structures: C, gray; H, white; O, red; S, yellow; F, light cyan. (**b**) Interaction energy of CO_2_/water adsorption on LF1 molecules with different geometrical configurations.

To systematically elucidate the site-specific affinities of the low-fluorinated surfactants toward both supercritical carbon dioxide (sc-CO_2_) and water, quantum chemical calculations based on Density Functional Theory (DFT) were first performed using the LF1 monomer as a representative model. Various initial orientations of a single CO_2_ or H_2_O molecule interacting with different functional groups of LF1 were optimized to obtain the global energy-minimized conformations, as presented in Figure 1. The lowest-energy optimized structures were categorized into four representative configurations: F-C-1, F-C-2, F-W-1, and F-W-2. Specifically, F-C-1 and F-W-1 illustrate the localized interactions of the fluorinated hydrophobic tail with a single CO_2_ molecule and a single H_2_O molecule, respectively. In contrast, F-C-2 and F-W-2 capture the coordinate configurations of the hydrophilic sulfonate headgroup with CO_2_ and H_2_O, respectively.

Given that fluorine atoms are well-documented to enhance surfactant solubility and stability in carbon dioxide environments, the localized solvent affinities of the fluorinated tail were quantified and compared. The calculated binding energies (∆Ebinding) for these targeted configurations are summarized in Figure 1b, where a more negative ∆Ebinding value signifies a thermodynamically stronger intermolecular interaction [22]. As depicted in Figure 1b, the binding energy of the fluorinated tail with CO_2_ (F-C-1) is significantly more negative than its corresponding interaction with water (F-W-1). Conversely, the hydrophilic sulfonate headgroup exhibits a markedly higher binding affinity toward water (F-W-2) compared to CO_2_ (F-C-2). These quantitative energetics clearly demonstrate that the fluorinated tail imparts a strong localized CO_2_-philicity, whereas the sulfonate headgroup governs the hydrophilic drive. This distinct spatial segregation of solvent affinities confirms the amphiphilic nature of the designed low-fluorinated surfactants, providing a thermodynamic basis for their interfacial orientation and organization in multicomponent sc-CO_2_/water systems.

**Figure 2:**
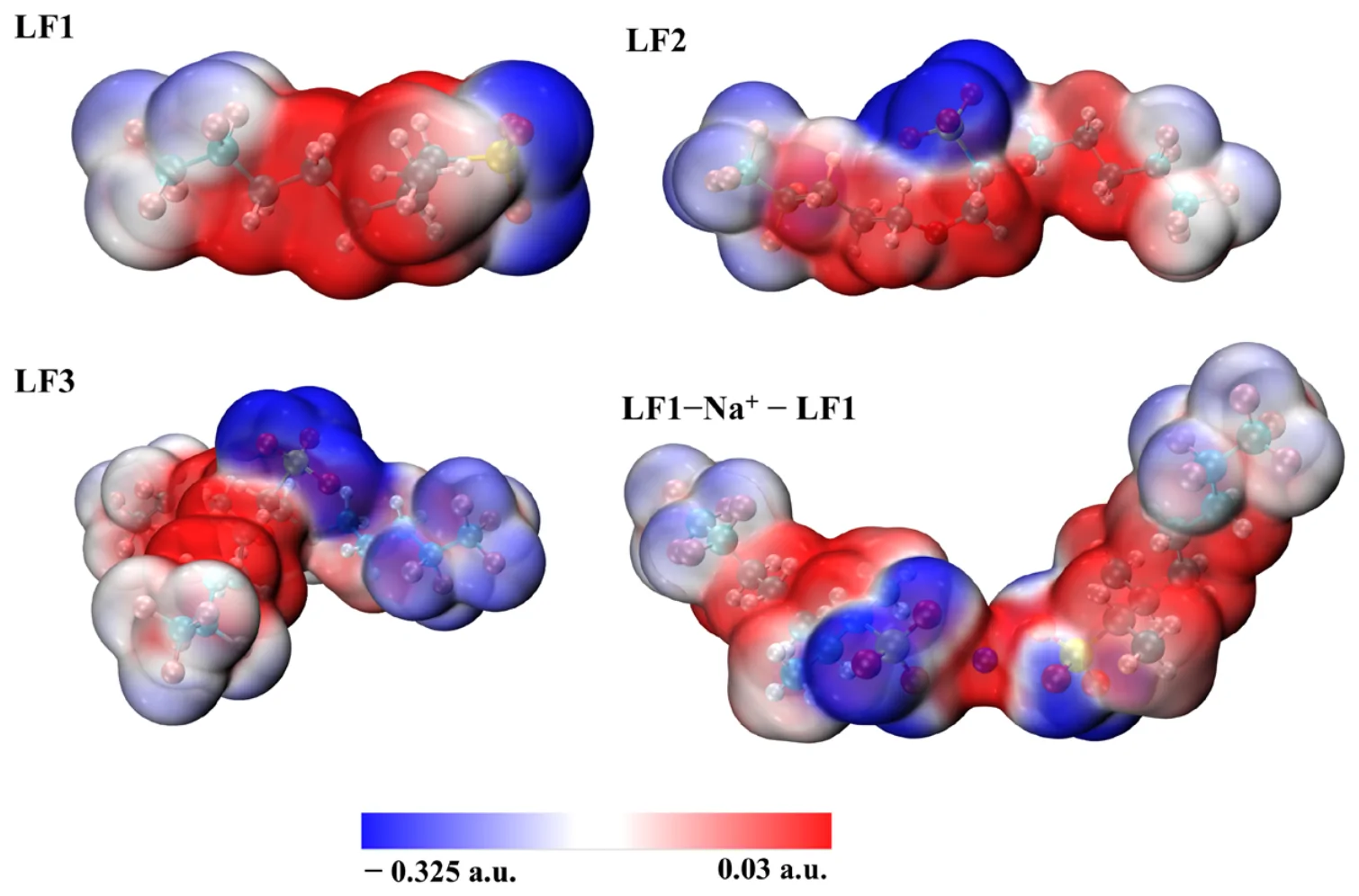
Stable geometries and MEP of LF series surfactants. Atom colors in the MEP: C, cyan; H, white; O, red; S, yellow; F, light pink.

The optimized molecular geometries and corresponding molecular electrostatic potential (MEP) distributions of the low-fluorinated surfactant series are illustrated in Figure 2. While the optimized LF1 and LF2 monomers adopt highly extended, linear backbone conformations, the three-tailed LF3 variant exhibits a distinct “claw-like” tetrahedral geometry. This steric folding is primarily driven by intramolecular steric hindrance and electrostatic repulsion among the closely packed hydrophobic tail branches. The topological distribution of the MEP plays a fundamental role in governing intermolecular recognition, molecular orientation, and local aggregation/packing of surfactant molecules [23]. Thermodynamically, regions exhibiting positive electrostatic potentials tend to align with negative electrostatic domains to minimize local electrostatic repulsion, thereby reducing the total free energy and favoring a stable associated state. The absolute magnitude of the local electrostatic potential directly correlates with the binding strength of electrostatic interactions and hydrogen bonding. For the LF1 monomer, the electrostatic potential is predominantly negative across the molecular envelope, with the most intense negative regions localized around the anionic sulfonate headgroup (-SO_3_^−^) and the highly electronegative fluorine atoms along the tail, reflecting the overall anionic character of the surfactant.

Intriguingly, in the presence of sodium counterions (Na^+^), the sulfonate headgroups of two adjacent LF1 monomers coordinate tightly around a central Na^+^ cation (as depicted in Figure 2). This coordinate behavior confirms that the counterions exert a strong electrostatic screening effect that suppresses headgroup-headgroup repulsion. Consequently, the Na^+^ cations act as localized charge-bridging agents that facilitate and stabilize intermolecular association and packing among the low-fluorinated surfactants.

### 2.2. Interfacial Behavior of Low-Fluorinated Surfactants at the sc-CO_2_/Water Interface

#### 2.2.1. Interfacial Organization and Density Distribution Profiles

**Figure 3:**
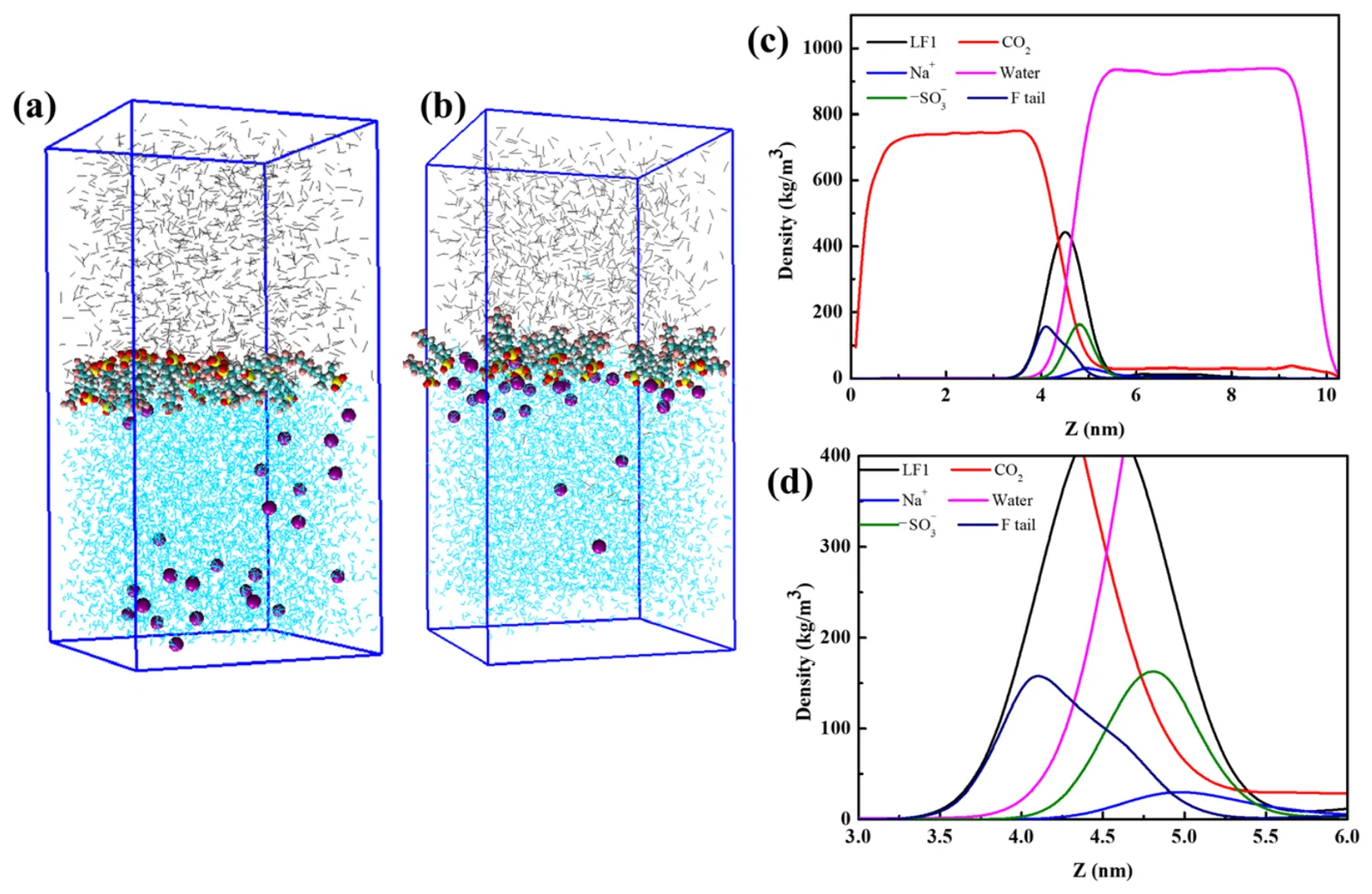
Simulation configuration of LF1 surfactant molecules in the sc-CO_2_/water system. (**a**) 0 ns and (**b**) 20 ns. Water and sc-CO_2_ molecules are displayed in blue and gray. Atom coloring scheme for LF: S, yellow, F, pink, O, red, and C, cyan. (**c**) Density profiles of the components along the Z-direction in the LF1 system. (**d**) Enlarged view of the density profiles of the components along the Z-direction in the LF1 system.

**Figure 4:**
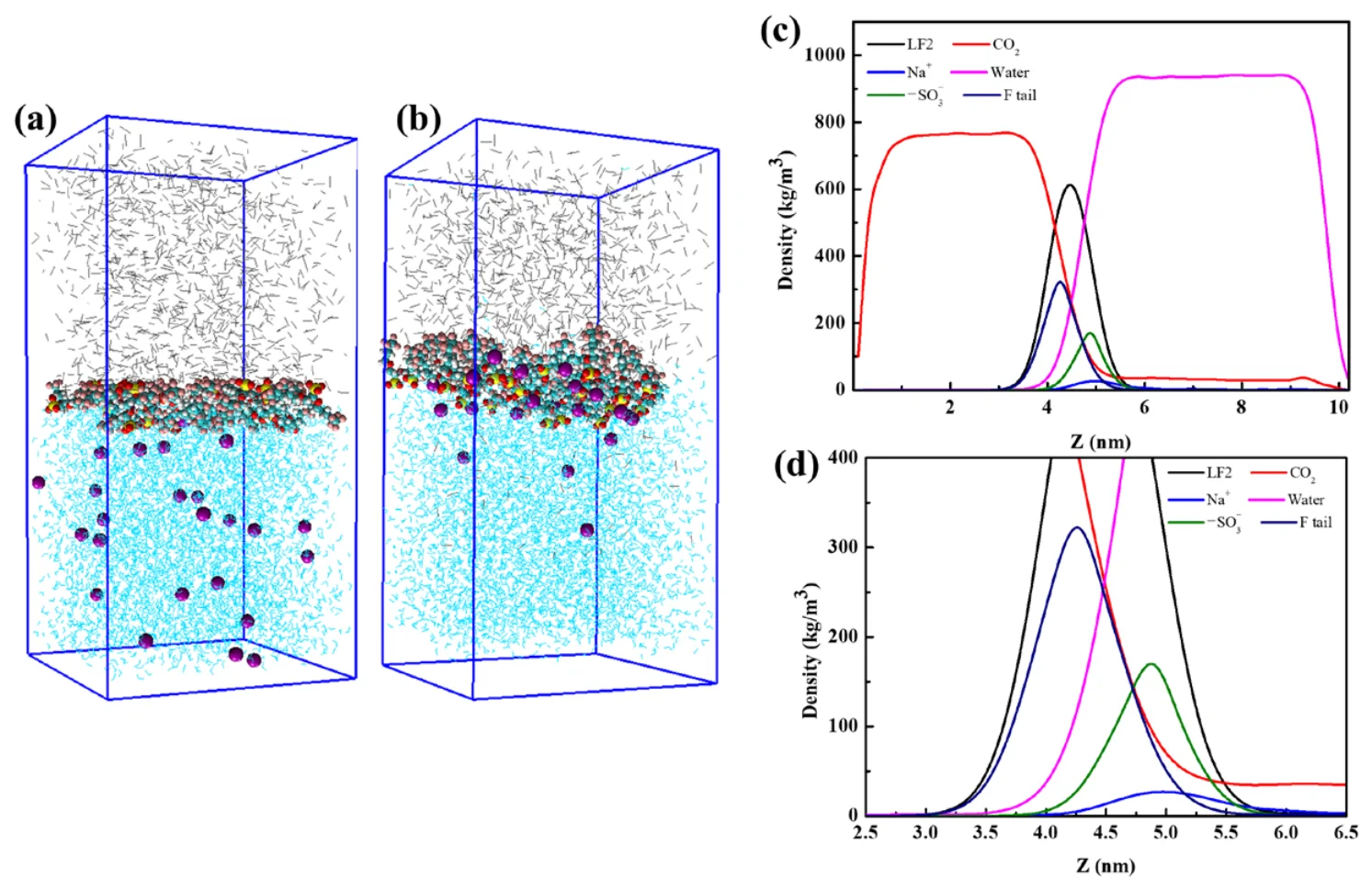
Simulation configuration of LF2 surfactant molecules in the sc-CO_2_/water system. (**a**) 0 ns, (**b**) 20 ns. (**c**) Density profiles of the components along the Z-direction in the LF2 system. (**d**) Enlarged view of the density profiles of the components along the Z-direction in the LF2 system.

**Figure 5:**
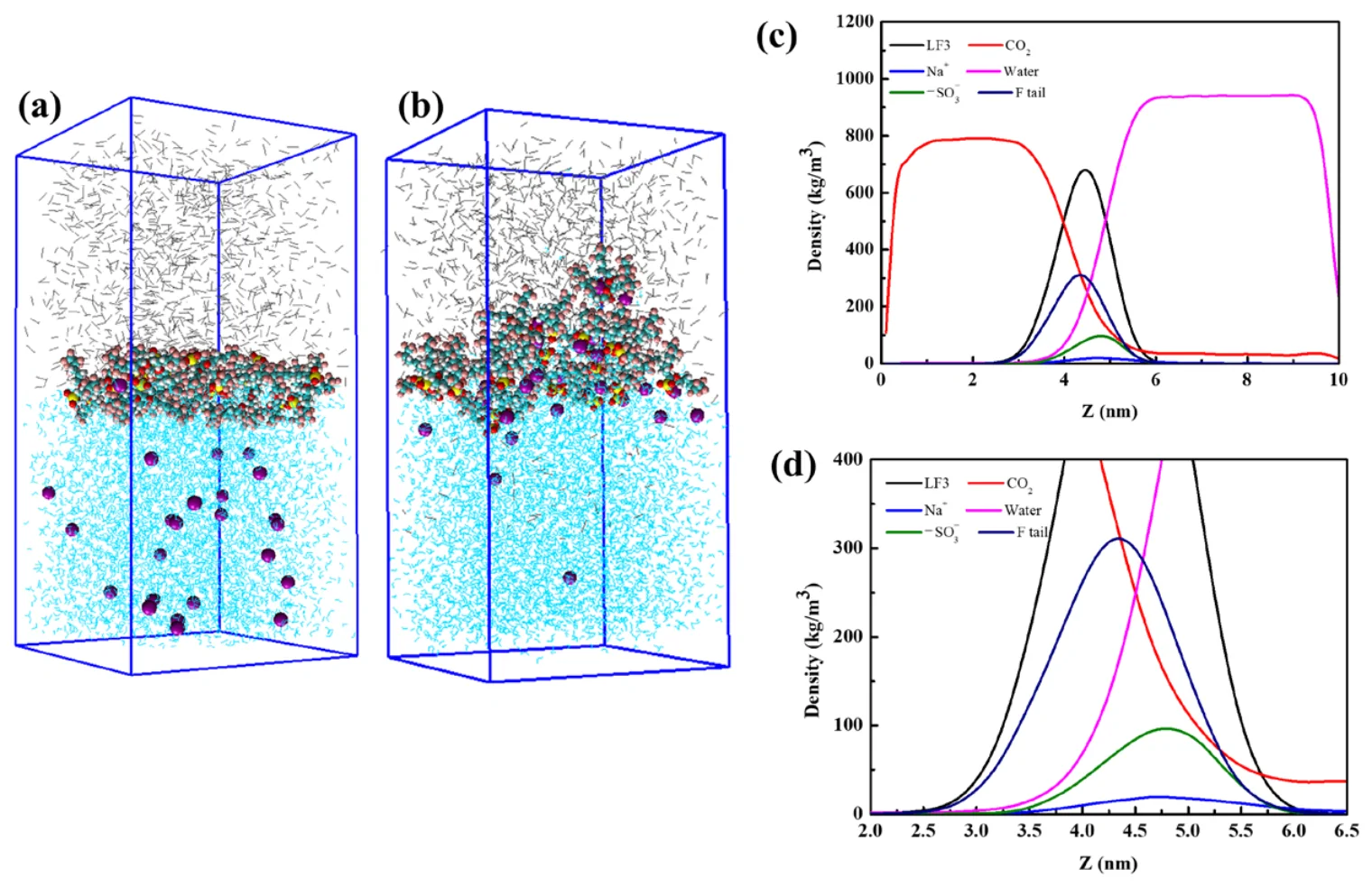
Simulation configuration of LF3 surfactant molecules in the sc-CO_2_/water system. (**a**) 0 ns, (**b**) 20 ns. (**c**) Density profiles of the components along the Z-direction in the LF3 system. (**d**) Enlarged view of the density profiles of the components along the Z-direction in the LF3 system.

Conventional surfactants, typically consisting of hydrophilic headgroups and hydrophobic tail chains, possess a thermodynamic tendency to adopt preferred orientations at oil–water interfaces. Analogously, the designed low-fluorinated surfactants—incorporating both hydrophilic and sc-CO_2_-philic moieties—are expected to exhibit preferential interfacial organization at the sc-CO_2_/water boundary. Variations in the balance of hydrophilic and CO_2_-philic characters among LF1, LF2, and LF3 are therefore expected to produce distinct interfacial configurations and packing states. Explicit-solvent molecular dynamics (MD) simulations were performed to elucidate the molecular conformations and spatial distributions of these three surfactants at the sc-CO_2_/water interface, with results displayed in Figure 3, Figure 4 and Figure 5. Using the LF1 system as a representative model, the surfactant molecules were initially positioned at the established sc-CO_2_/water interface, while the Na+ counterions were introduced into the aqueous phase. As the MD trajectory progressed, the LF1 molecules reorganized within the interfacial transition region and gradually developed a heterogeneous surfactant-rich layer. In the relaxed interfacial configuration, the hydrophilic sulfonate headgroups are oriented toward the aqueous phase, whereas the low-fluorinated tails project into the sc-CO_2_ phase. Concurrently, the Na^+^ counterions localize in close proximity to the anionic sulfonate (-SO_3_^−^) headgroups.

This spatial organization is quantitatively confirmed by the 1D atomic number density profiles, which show the density peaks of the fluorinated tails, the sulfonate groups, and the Na^+^ ions aligned at z = 4.1 nm, 4.8 nm, and 5.1 nm, respectively, in perfect agreement with the simulation snapshots. The number density profiles for all three systems (LF1, LF2, and LF3) consistently place their primary surfactant peaks at the liquid–fluid boundary, proving that all three variants act as active interfacial agents. However, differences in tail branching modify the structural packing of the interfacial film, leading to distinct film thicknesses. The interfacial film thickness (d_film_) for each system was calculated using the 10–90% density profile criterion [24]:

(1) $$d_{film}=z_{90\%}-z_{10\%}$$

where z(90%) and z(10%) represent the coordinates where the local density of the surfactant phase is 90% and 10% of its bulk-equivalent peak value, respectively. The computed thicknesses for the LF1, LF2, and LF3 monolayers were 2.08 nm, 2.49 nm, and 3.12 nm, respectively. The significantly greater thickness of the LF3 interfacial film is attributed to its three-tailed branched architecture, which promotes intermolecular entanglement and steric overlapping among adjacent hydrophobic tails, expanding the interfacial boundary.

#### 2.2.2. Radial Distribution Functions (RDFs) and Local Microstructures

**Figure 6:**
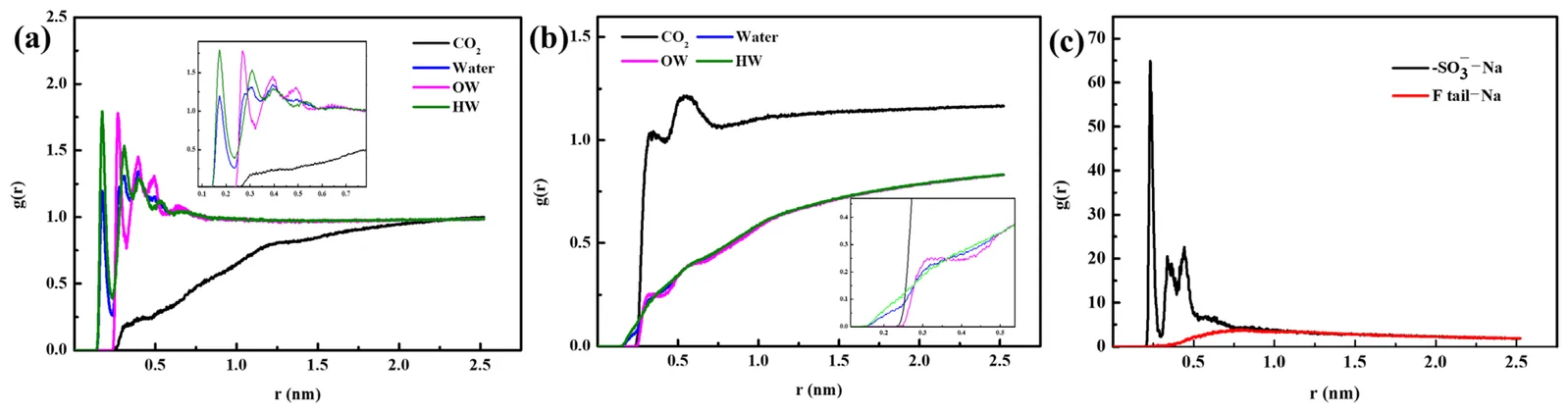
(**a**) RDFs between -SO_3_^−^ and the components in the LF3 system, (**b**) RDFs between F tail and the components in the LF3 system, (**c**) RDFs between -SO_3_^−^ and F tail and Na^+^ in the LF3 system.

To quantitatively map the local solvation structures and coordination shells of the interfacial monolayer, radial distribution functions (g(r)) were calculated for the representative LF3 system. Figure 6a illustrates the RDFs of the hydrophilic -SO_3_^−^ headgroup with water and CO_2_ molecules. The -SO_3_^−^-water g(r) curve shows a sharp, intense first peak at r = 0.18 nm, corresponding to the primary hydration shell. A secondary, well-defined solvation peak appears at r = 0.25 nm, indicating a highly organized, dense local clustering of water molecules around the sulfonate headgroup. Conversely, the -SO_3_^−^-CO_2_ g(r) profile displays no discernable peaks within a 1.0 nm radius, confirming that CO_2_ molecules are strongly excluded from the highly hydrophilic headgroup region, which aligns with the density profile results.

The coordination of the fluorinated tails with the solvent phases is plotted in Figure 6b. The tail-CO_2_ g(r) profile features prominent peaks at r = 0.25 nm and 0.55 nm, reflecting a highly concentrated local solvation shell of sc-CO_2_ surrounding the low-fluorinated aliphatic chains. In contrast, the tail-water g(r) curve remains flat without any peaks up to 1.0 nm, showing that water molecules are completely excluded from the fluorinated hydrophobic domain.

Furthermore, the RDFs of both the sulfonate headgroups and the fluorinated tails with the Na^+^ counterions were evaluated (Figure 6c). A sharp, high-intensity peak in the -SO_3_^−^-Na^+^ g(r) curve indicates strong coordinate-like electrostatic binding. In contrast, the tail-Na^+^ g(r) curve exhibits no peak, confirming that the counterions are localized near the hydrophilic heads. This positioning allows the counterions to act as electrostatic bridges, suppressing headgroup repulsion and stabilizing the tightly packed interfacial monolayer.

#### 2.2.3. Molecular Mobility, Diffusion Coefficients, and Interaction Energetics

**Figure 7:**
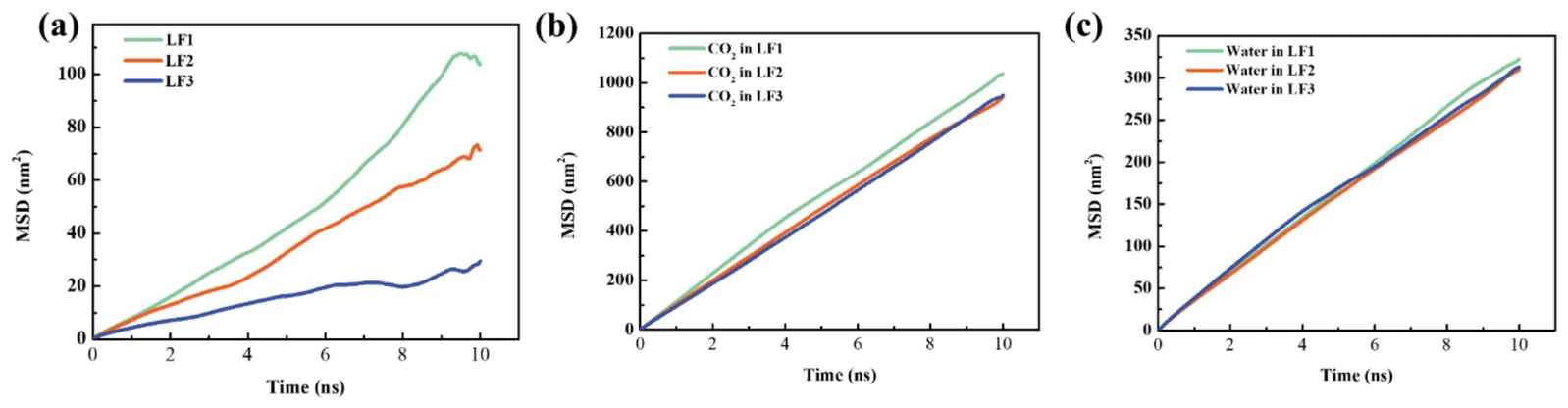
MSD curves of various components in different systems as a function of time: (**a**) surfactant in each system, (**b**) CO_2_ in each system, (**c**) water in each system.

To evaluate the kinetic stability of the interfacial systems, the mean square displacement (MSD) was monitored as a function of simulation time for the surfactant, CO_2_, and water phases (Figure 7). All MSD curves exhibit a linear regime over the production phase, demonstrating that the systems reached stable diffusion equilibria. The diffusion coefficients (D) of the individual components were extracted from the Einstein relation and are summarized in Table 1.

The translational diffusion coefficient of the LF1 monomer is significantly higher than those of the other two variants, indicating high molecular mobility. Conversely, LF3 exhibits the lowest diffusion rate. This reduced mobility is attributed to its three-tailed branched geometry, which increases intermolecular friction and tail-tail entanglement, restricting translational motion within the monolayer. The reduced mobility of LF3 likely arises from the combined effects of its increased molecular size/mass and its three-tailed branched architecture. Notably, The diffusion coefficients of water and CO_2_ exhibit an overall decreasing trend from LF1 to LF3. However, the values for LF2 and LF3 are relatively close, indicating that the additional effect of the third hydrophobic tail on bulk solvent mobility is limited. The introduction of additional hydrophobic tails modifies interfacial packing, steric interactions, and local molecular confinement. The transition from LF1 to LF2 produces a pronounced change in the interfacial environment, whereas the further transition from LF2 to LF3 primarily enhances the compactness and stability of the surfactant monolayer itself. Since the calculated solvent diffusion coefficients are averaged over both interfacial and bulk-like molecules, the stronger local confinement in LF3 results in only a modest further decrease in the overall water and CO_2_ diffusion coefficients.

**Figure 8:**
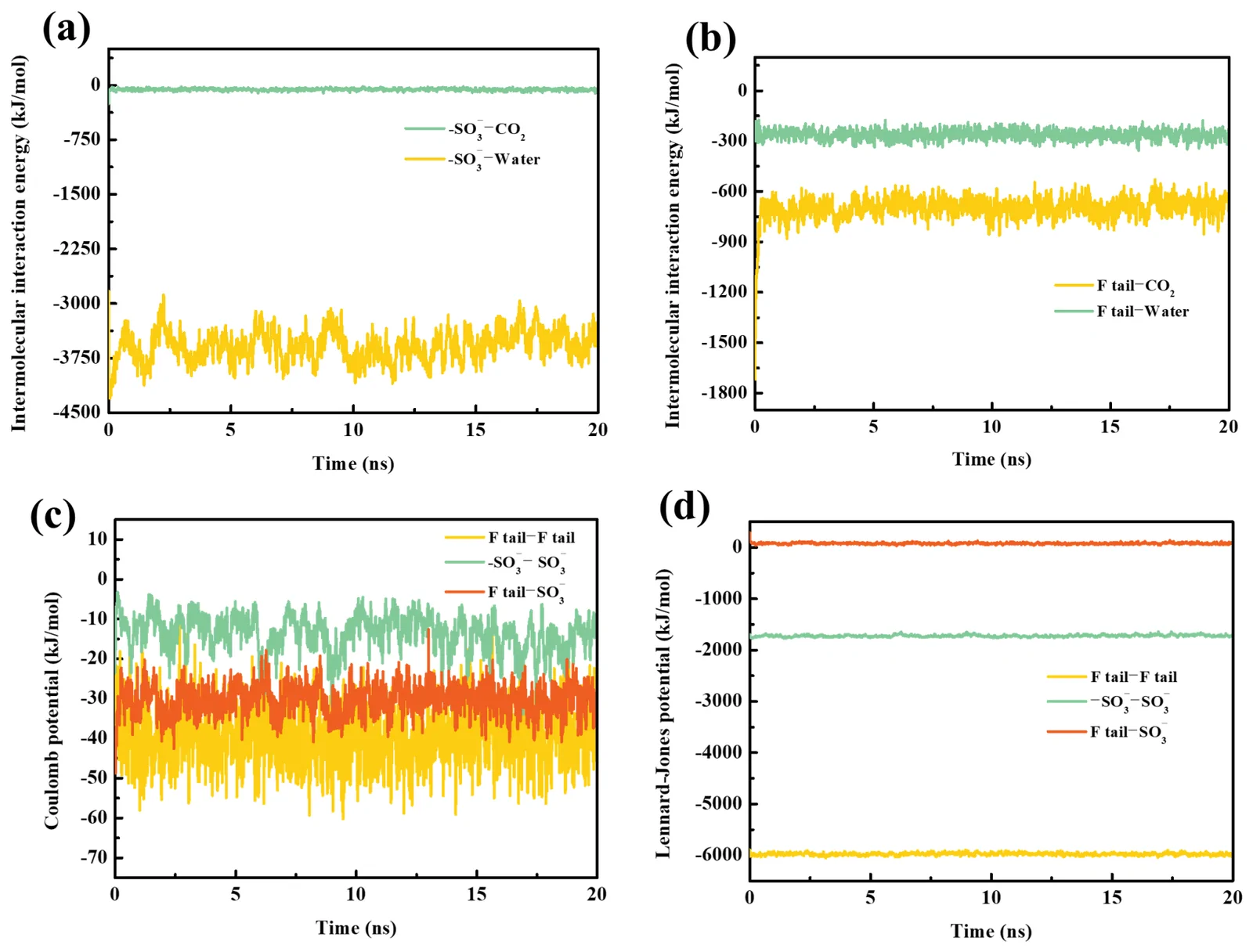
Intermolecular interaction energy between the -SO_3_^−^ (**a**) and F tail (**b**)with CO_2_ and water, respectively. Coulomb potential (**c**) and Lennard-Jones potential (**d**) between LF3 molecules.

To validate this thermodynamic behavior, the non-bonded interaction energies between the individual components were calculated. As shown in Figure 8, the interaction energy between the sulfonate headgroup and water is significantly more negative than its interaction with CO_2_. Similarly, the fluorinated tail interacts more strongly with CO_2_ than with water.

To clarify the molecular interactions responsible for the organization and stabilization of the surfactant monolayer, the electrostatic and van der Waals (vdW) interaction energies among adjacent surfactant molecules were calculated separately for the tails and headgroups. As shown in Figure 8c,d, the electrostatic interaction between adjacent fluorinated tails is minor, whereas their cohesive vdW interaction energy is highly negative. A similar trend is observed for the sulfonate headgroups, where electrostatic repulsion is shielded by the Na^+^ ions, allowing cohesive vdW forces to dominate. This strong, cooperative vdW interaction promotes tight molecular packing and stabilizes the cohesive interfacial film.

Meanwhile, the adequacy of the 20 ns simulation time was evaluated using both energetic and dynamical criteria. As shown in Figure 8, after the initial relaxation period the system energy fluctuated around a stable mean value without systematic drift, indicating energetic convergence. In addition, the MSD curves used to determine the diffusion coefficients reported in Figure 7 entered a stable linear regime at long times. According to the Einstein relation, this linear MSD behavior corresponds to the normal diffusive regime and is required for reliable extraction of self-diffusion coefficients. The simultaneous stabilization of the energy and MSD behavior was therefore used to confirm that the systems had reached a statistically stable state within the 20 ns simulation period.

#### 2.2.4. Conformation Compactness and Interfacial Formation Energy (IFE)

To further evaluate the structural stability and packing of the interfacial monolayers, the mean radius of gyration 〈Rg〉 of the surfactants was calculated. Typically, a smaller 〈Rg〉 value corresponds to a more compact molecular conformation. As presented in Table 2, the average radius of gyration follows the order of ${\langle Rg\rangle }_{LF3}<{\langle Rg\rangle }_{LF2}<{\langle Rg\rangle }_{LF1}$. The minimized gyration of LF3 indicates that its branched tails fold into a compact conformation, leading to a highly cohesive and densely packed monolayer.

Finally, the thermodynamic stability of the surfactant-covered interface was quantified by calculating the Interfacial Formation Energy (IFE):

(2) $$IFE=\frac{E_{total}-(E_{LF}\times n+E_{ref})}{n}$$

where Etotal is the potential energy of the fully equilibrated ternary surfactant-solvent system, Eref is the reference energy of the bare, surfactant-free sc-CO_2_/water interface, n is the number of interfacial surfactant molecules, and ELF is the potential energy of an isolated surfactant monomer calculated in a separate vacuum simulation. A more negative IFE, with a larger absolute magnitude, indicates greater energetic stabilization of the surfactant-covered interface and a more stable interfacial film, whereas a smaller absolute value reflects comparatively lower interfacial stability [25].

The computed IFE values are summarized in Table 2. Across all systems, the LF3-stabilized interface yields the most negative IFE value. This energetic preference indicates that LF3 forms the most stable cohesive interfacial monolayer among the three systems. This highly stable interfacial structure provides a favorable molecular environment for the formation of robust associative structures in bulk supercritical CO_2_.

### 2.3. Synergistic Blending of Low-Fluorinated Surfactants and Non-Fluorinated Co-Surfactants

To systematically mitigate the environmental footprint and cost of fluorinated materials, a binary composite formulation was designed by blending the low-fluorinated surfactant with a fluorine-free branched hydrocarbon co-surfactant (ZBJ). Explicit-solvent molecular dynamics simulations were conducted to explore the interfacial organization and synergistic packing of this blended system. After a 20.0 ns production run, the system reached a statistically stable interfacial state.

**Figure 9:**
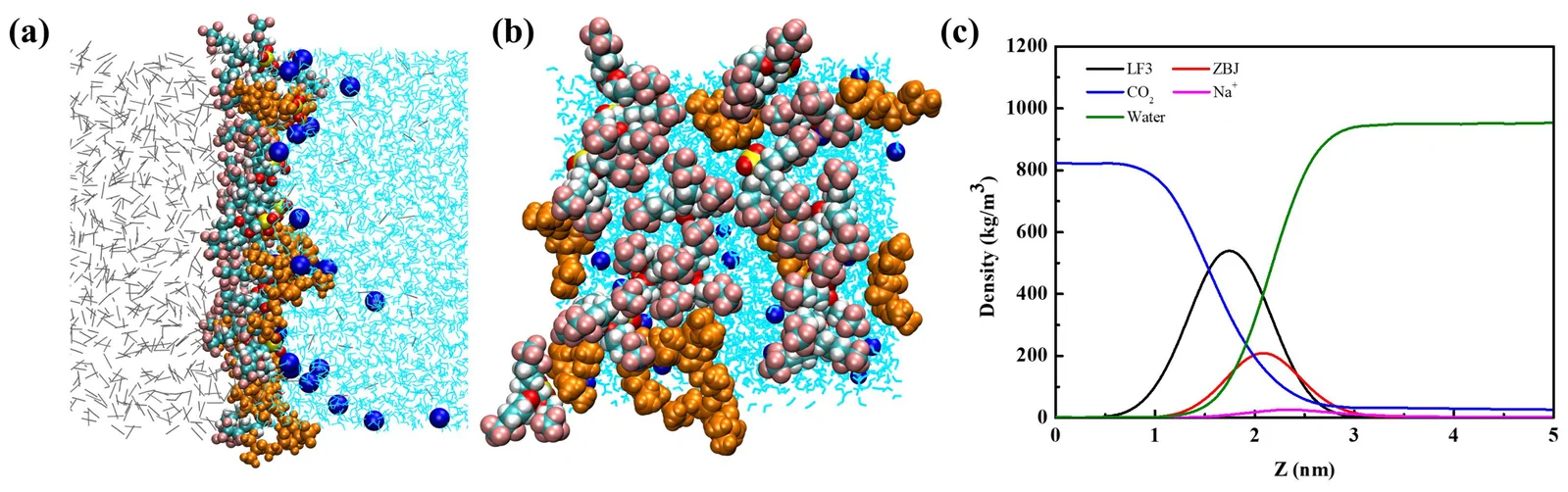
Simulation configuration at the end (20 ns) of compounding system: (**a**) side view, (**b**) front view. Water and sc-CO_2_ molecules are displayed in blue and gray. Atom coloring scheme for LF3: S, yellow, F, pink, O, red, and C, cyan, ZBJ: orange, (**c**) Density profiles of the components along the z-direction in compounding system.

The equilibrium conformations of the blended LF3/ZBJ system are illustrated in Figure 9a,b. Analysis of the cross-sectional side view reveals that the ZBJ co-surfactant molecules co-organize with the LF3 monomers at the sc-CO_2_/water interface. From the corresponding top view, the compact, branched tails of ZBJ occupy structural packing voids (interstitial free volume) between the bulky claw-like LF3 molecules. This steric complementarity enhances the local packing density, yielding a cohesive and stable mixed interfacial monolayer.

To characterize the spatial distribution of each component, the 1D atomic number density profiles along the z-axis were computed (Figure 9c). The density peak of the ZBJ co-surfactant is positioned precisely at the liquid–fluid boundary between the sc-CO_2_ and aqueous phases. Notably, the maximum density coordinate of ZBJ is slightly shifted toward the water bulk phase relative to the primary peak of LF3. This positional shift is primarily attributed to the non-fluorinated chemical structure of the ZBJ tail, which lacks the highly CO_2_-philic fluorine atoms and thus experiences a weaker thermodynamic pull into the bulk sc-CO_2_ phase compared to LF3.

To evaluate the thermodynamic stability of the mixed monolayer, the Interfacial Formation Energy of the binary surfactant mixture (IFE) was calculated using the following thermodynamic relation:

(3) $$IFE=\frac{E_{total}-(E_{LF}\times m+E_{ZBJ}\times n+E_{ref})}{\left(m+n\right)}$$

where Etotal represents the total potential energy of the fully equilibrated mixed ternary system; Eref is the reference potential energy of the pristine, surfactant-free sc-CO_2_/water interface; m and n denote the stoichiometric quantities of interfacial LF and ZBJ molecules within the mixed monolayer (m = 25, n = 25), respectively; and ELF and EZBJ correspond to the potential energies of isolated, single LF and ZBJ monomers evaluated in independent vacuum simulations.

The calculated IFE for the LF3/ZBJ binary formulation was determined to be −342.07 ± 3.49 kJ/mol. This value is thermodynamically more negative than that of the single-component LF3 monolayer system (−330.30 ± 2.78 kJ/mol). The increased energy release indicates stronger energetic stabilization of the binary interfacial layer and supports the formation of a cohesive, stable mixed monolayer.

In summary, these findings demonstrate that incorporating a branched hydrocarbon co-surfactant can successfully minimize the overall fluorine content of the formulation without compromising the mechanical and thermodynamic stability of the sc-CO_2_/water interface. This structural synergy provides a valuable paradigm for the rational design of highly sustainable, low-fluorine sc-CO_2_ thickener systems.

## 3. Materials and Methods

### 3.1. Molecular Model Construction and Force Field Parameters

Three low-fluorinated hydrocarbon surfactants characterized by distinct hydrophobic tail-branching architectures—comprising one, two, and three tail branches—were designed and designated as LF1, LF2, and LF3, respectively. To further lower the overall fluorine content of the formulation, a fluorine-free branched co-surfactant (denoted as ZBJ) was designed to act as a synergetic agent in mixed surfactant systems. The structures of the surfactants are shown in Table 3.

The OPLS-AA force field [26] was selected for the low-fluorine surfactants and the co-surfactant because all-atom OPLS-type models have been widely applied to fluorinated compounds and fluorinated surfactants and have reproduced experimentally accessible transport and structural properties in related systems [27,28]. Water was represented by the SPC/E model, which has an established record in condensed-phase simulations and has been used in combination with OPLS-AA for fluorinated aqueous systems [29]. In addition, SPC/E has been specifically combined with the EPM2 CO_2_ model in previous studies of water-CO_2_ mixtures [30]. All initial coordinates and multi-component boxes were packed using the Packmol (version 21.0.0) [31] program under strict minimum-distance constraints to prevent unphysical atomic overlaps.

### 3.2. Simulation Systems and Boundary Conditions

To investigate the interfacial behavior of the low-fluorinated surfactants at the fluid–fluid interface, an amorphous sc-CO_2_ phase box with dimensions of 5.0 × 5.0 × 5.0 nm^3^ was constructed, containing exactly 4120 sc-CO_2_ molecules. This molecule count was derived from the bulk phase density of pure sc-CO_2_ (ρ = 725.0 kg/m^3^) under reservoir conditions of T = 358.15 Kand P = 30.0 MPa according to the National Institute of Standards and Technology (NIST) Chemistry WebBook [32]. Concurrently, an aqueous phase box of identical dimensions (5.0 × 5.0 × 5.0 nm^3^) containing 5000 H_2_O molecules was constructed, which corresponds to the liquid water density under the same thermodynamic state point (ρ = 981.0 kg/m^3^ at 358.15 K and 30.0 MP).

To focus specifically on the relaxation, packing, and orientation of a pre-existing surfactant-covered interface rather than on bulk-to-interface adsorption kinetics, a total of 25 surfactant molecules were initially positioned symmetrically at the established 25.0 nm^2^ liquid–fluid boundary. This yielded a ternary sc-CO_2_/LF/water simulation cell with final dimensions of 5.0 × 5.0 × 10.0 nm^3^ operated under three-dimensional (3D) periodic boundary conditions. Because the LF surfactants contain an anionic sulfonate headgroup, a stoichiometric amount of Na^+^ counterions was explicitly introduced into each MD system to maintain overall electrical neutrality.

To quantify the site-specific interactions and binding affinities between the surfactant functional groups and the solvent molecules (CO_2_ and H_2_O), quantum chemical calculations were executed using the Gaussian 16 [33]. Initial configurations of a single CO_2_ or H_2_O molecule adsorbed onto distinct functional moieties of the LF1 monomer were optimized without symmetry constraints using the hybrid B3LYP functional coupled with the def2-SVP basis set [22].

Subsequent single-point energy (SPE) evaluations of the optimized geometries were performed at a higher level of theory using the B3LYP functional [34] set and the quadruple-zeta def2-QZVP basis set [35]. The core binding energy (∆E_binding_) was determined via the standard supramolecular energy approach:

(4) $$\Delta E=E_{total}-E_{surfac\tan t}-E_{CO_{2}/water}$$

where $E_{total}$, $E_{surfac\tan t}$ and $E_{CO_{2}/water}$ represent the total electronic energies of the optimized surfactant-adsorbate complex, the isolated surfactant monomer, and the isolated solvent molecule (CO_2_ or H_2_O), respectively. Electrostatic potential (ESP) maps were generated based on the derived electron density topologies to visualize local charge distributions.

All explicit-solvent MD simulations and trajectory post-processing were executed via GROMACS (version 2019.6) [36,37]. The potential energy of all initial configurations was minimized using the steepest descent algorithm followed by the conjugate gradient method until the maximum atomic force tolerance (Fmax) dropped below 1000 kJ/mol/nm.

Subsequently, a 20 ns production trajectory was run in the canonical (NVT) ensemble at a constant temperature of 358.15 K. Thermal regulation was enforced using the velocity-rescaling (V-rescale) thermostatting algorithm [38] with a coupling time constant of 0.1 ps. All covalent bonds involving hydrogen atoms were constrained using the Linear Constraint Solver (LINCS) algorithm [39], enabling an integration time step (dt) of 2.0 fs. Short-range Lennard-Jones (vdW) interactions were truncated at a directional cut-off radius of 1.2 nm. Long-range electrostatic forces were computed in reciprocal space using the Particle Mesh Ewald (PME) method [40] with a grid spacing of 0.12 nm and a fourth-order cubic interpolation under three-dimensional periodic boundary conditions. The final 10 ns of simulation trajectories were used for calculating the diffusion coefficients of each component. The simulation data from the last 5 ns of the trajectory were adopted for other subsequent result analysis. Each system was simulated in triplicate, and the average values were taken for the analytical results. Visualization and trajectory diagnostic rendering were performed using Visual Molecular Dynamics VMD software (version 1.9.3) [41].

**Figure 10:**
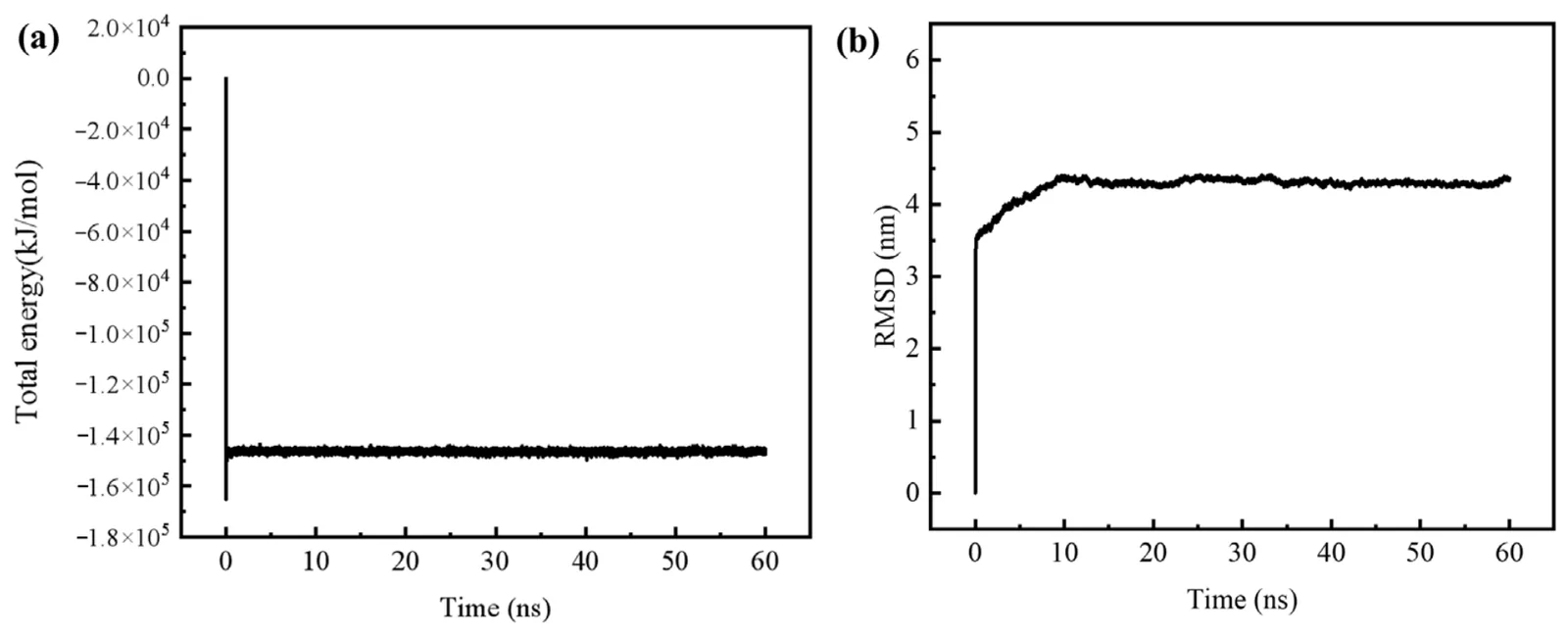
(**a**) Total energy of the system as a function of time; (**b**) RMSD of LF3 as a function of time.

To further assess the adequacy of the simulation time, the LF3 system was extended from 20 ns to 60 ns. The total energy showed no systematic drift after initial relaxation, and the RMSD reached a stable plateau after approximately 10 ns (Figure 10) and remained stable up to 60 ns. These results indicate that the major energetic and structural relaxation had been completed before the 15–20 ns interval used for statistical analysis.

## 4. Conclusions

In this work, a series of low-fluorinated surfactants (LF1–3) containing one, two, and three hydrophobic tails with terminal fluorine atoms was designed. Their molecular behavior and interfacial organization at the sc-CO_2_/water interface were systematically investigated using density functional theory (DFT) and molecular dynamics (MD) simulations. The main conclusions are as follows: DFT calculations and molecular electrostatic potential (MEP) distributions confirmed the amphiphilic nature of LF1–3. The fluorinated tails show preferential affinity toward CO_2_, whereas the sulfonate headgroups interact more strongly with water, providing the thermodynamic basis for interfacial orientation. MD simulations demonstrated that the surfactants, initially positioned at the interface, undergo interfacial reorganization and orientational ordering, with the fluorinated tails extending into the sc-CO_2_ phase and sulfonate groups oriented toward the water phase. Among them, the three-tailed, claw-like LF3 surfactant exhibits the most stable and densely packed interfacial film. Moreover, synergistic blending with the fluorine-free co-surfactant (ZBJ) optimizes molecular packing by filling interstitial voids at the interface, leading to high energetic stability of the mixed interfacial monolayer. Collectively, these findings establish a theoretical foundation for the rational design of low-fluorine surfactant systems with tailored interfacial properties, offering molecular-level guidance for the development of sc-CO_2_ formulations.

## Figures and Tables

**Table 1:** Diffusion coefficients of the components in the different systems.

System	D, 10^−5^ cm^2^/s		
	Surfactant	CO_2_	Water
LF1	1.8024 ± 0.4103	16.8557 ± 0.6448	5.4543 ± 0.0946
LF2	1.2393 ± 0.3269	15.9030 ± 0.5886	5.0615 ± 0.4768
LF3	0.4003 ± 0.2985	15.3789 ± 0.6330	4.9933 ± 0.7002

**Table 2:** Interface formation energy (IFE) in the different system and mean radius of gyration 〈Rg〉 of the surfactants.

System	Energy, kJ/mol				〈Rg〉,nm
	Total	CO_2_/Water	Surfactant	IFE	
LF1	−142,506	−129,637	−225.43	289.34 ± 2.36	2.47 ± 0.08
LF2	−139,578	−129,637	−89.57	308.07 ± 1.89	2.33 ± 0.05
LF3	−136,390	−129,637	60.18	330.30 ± 3.68	2.25 ± 0.06

**Table 3:** Molecular structure and 3D model of LF series surfactants and cosurfactant. Atom colors: C, gray; H, white; O, red; S, yellow; F, light cyan.

Surfactant	Structure	3D Model
LF1	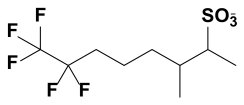	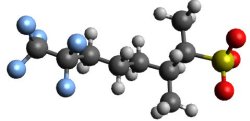
LF2	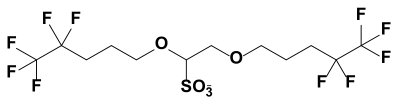	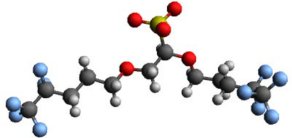
LF3	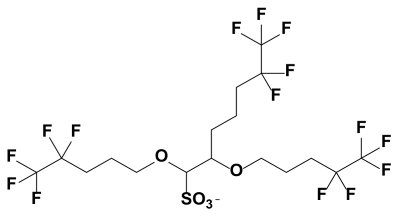	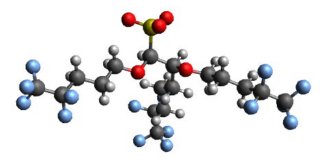
ZBJ	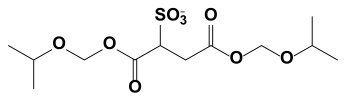	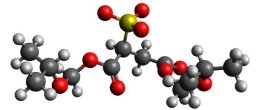

## Data Availability

The data will be made available upon request.
